# Spin echo versus stimulated echo diffusion tensor imaging of the in vivo human heart

**DOI:** 10.1002/mrm.25998

**Published:** 2015-10-07

**Authors:** Constantin von Deuster, Christian T. Stoeck, Martin Genet, David Atkinson, Sebastian Kozerke

**Affiliations:** ^1^ Imaging Sciences and Biomedical EngineeringKing's College LondonLondonUnited Kingdom; ^2^ Institute for Biomedical EngineeringUniversity and ETH ZurichZurichSwitzerland; ^3^ Centre for Medical ImagingUniversity College LondonLondonUnited Kingdom

**Keywords:** myocardial fiber architecture, cardiac diffusion tensor imaging, STEAM, spin‐echo, motion compensation, signal‐to‐noise ratio

## Abstract

**Purpose:**

To compare signal‐to‐noise ratio (SNR) efficiency and diffusion tensor metrics of cardiac diffusion tensor mapping using acceleration‐compensated spin‐echo (SE) and stimulated echo acquisition mode (STEAM) imaging.

**Methods:**

Diffusion weighted SE and STEAM sequences were implemented on a clinical 1.5 Tesla MR system. The SNR efficiency of SE and STEAM was measured (b = 50–450 s/mm^2^) in isotropic agar, anisotropic diffusion phantoms and the in vivo human heart. Diffusion tensor analysis was performed on mean diffusivity, fractional anisotropy, helix and transverse angles.

**Results:**

In the isotropic phantom, the ratio of SNR efficiency for SE versus STEAM, SNR_t_(SE/STEAM), was 2.84 ± 0.08 for all tested b‐values. In the anisotropic diffusion phantom the ratio decreased from 2.75 ± 0.05 to 2.20 ± 0.13 with increasing b‐value, similar to the in vivo decrease from 2.91 ± 0.43 to 2.30 ± 0.30. Diffusion tensor analysis revealed reduced deviation of helix angles from a linear transmural model and reduced transverse angle standard deviation for SE compared with STEAM. Mean diffusivity and fractional anisotropy were measured to be statistically different (*P* < 0.001) between SE and STEAM.

**Conclusion:**

Cardiac DTI using motion‐compensated SE yields a 2.3–2.9× increase in SNR efficiency relative to STEAM and improved accuracy of tensor metrics. The SE method hence presents an attractive alternative to STEAM based approaches. Magn Reson Med 76:862–872, 2016. © 2015 The Authors. Magnetic Resonance in Medicine published by Wiley Periodicals, Inc. on behalf of International Society for Magnetic Resonance in Medicine. This is an open access article under the terms of the Creative Commons Attribution License, which permits use, distribution and reproduction in any medium, provided the original work is properly cited.

## INTRODUCTION

The fiber architecture of the heart has significant influence on cardiac function, mechanical contraction and electrophysiology 1, 2, 3, 4, 5. The principal orientation of myofibers can be obtained from histological studies 6, 7, 8 or ex vivo 9, 10, 11 and in vivo 12, 13, 14, 15, 16, 17, 18, 19, 20, 21, 22, 23, 24 diffusion tensor imaging (DTI). While histological exams provide localized information on myocyte orientation with very high spatial resolution ex vivo, cardiac DTI allows assessment of myofiber aggregates noninvasively and in vivo. In agreement with histology, ex vivo DTI studies have demonstrated the characteristic circumferential alignment of myofibers with a distinct double helical pattern from endo‐ to epicardium 25, 26, 27. Fiber disarray and myocardial remodeling due to myocardial infarction and cardiomyopathies have been assessed by DTI methods both in animal and humans subjects 28, 29, 30, 31, 32, 33. Moreover, microstructural integrity of the myocardium has been described using mean diffusivity (MD) and fractional anisotropy (FA). Whereas mean diffusivity increased in myocardial infarction, FA was found to decrease 22, 28, 30, 34. These findings highlight the potential of in vivo cardiac DTI to allow for structural and functional tissue characterization in a range of relevant diseases.

In vivo cardiac DTI has primarily been performed using the stimulated echo acquisition mode (STEAM) 12, 13, 15, 17, 18, 20, 21, 22, 23, 33. Alternatively, spin‐echo (SE) imaging is feasible provided that motion compensated diffusion gradients are employed or dedicated postprocessing to account for motion‐induced signal loss is used 16, 24, 35, 36, 37, 38. Image formation is typically accomplished using echo planar imaging (EPI) or balanced steady‐state free precession imaging 39. Despite the advances in sequence design and data processing, DTI of the beating heart remains a challenging task due to low signal‐to‐noise‐ratio (SNR), off‐resonance artifacts, cardiac bulk motion, and myocardial strain.

In STEAM, diffusion encoding ranges across two consecutive heartbeats and hence the spatial position and shape of the myocardium are required to be identical in subsequent cardiac cycles. Accordingly, dedicated breath‐holding and navigator gating schemes are essential to suppress respiratory motion induced displacements. Alternatively, free‐breathing acquisition can be performed in combination with a dedicated navigator gating strategy and optional patient feedback system 18. The effect of myocardial strain during diffusion encoding using STEAM has to be considered by acquiring at so‐called “sweet spots” 15 in the cardiac cycle. Alternatively, separately acquired strain data 20, 23 may be used to correct for strain effects in the DTI data. A key advantage of STEAM over SE relates to the modest gradient hardware requirements as diffusion encoding takes place over a whole cardiac cycle and hence relatively low diffusion encoding gradient strengths are sufficient for adequate diffusion weighting.

With recent improvements in gradient hardware becoming widely available on clinical MR systems and dedicated diffusion gradient designs, diffusion weighted single‐shot spin‐echo (SE) sequences have become feasible for in vivo cardiac DTI. Several studies have shown that signal attenuation due to myocardial motion can be addressed successfully by incorporating motion compensated diffusion gradient waveforms 17, 19, 35, 36, 37, 38, 40, 41, 42, 43. Promising results of the in vivo human 38 and rat 42 heart using second and third order motion compensated DTI have been presented recently.

While image and data quality depend on many parameters including residual motion, off‐resonance and eddy‐current effects 44, the low SNR of cardiac DTI is a significant impediment to wider adoption of the technique in the clinic. Besides the need for patient feedback 18 and dedicated data postprocessing 23, 45, low scan time efficiency is a major reason for the small number of cardiac DTI studies on patients 46, 47, 48. Accordingly, a comparison of the available sequence approaches with regard to SNR and time efficiency is warranted to guide further improvements.

It is the objective of the present work to assess and compare SNR efficiency and diffusion metrics derived from cardiac DTI using acceleration‐compensated diffusion‐weighted SE and cardiac triggered STEAM in both phantoms and in the in vivo human heart.

## METHODS

**Figure 1:**
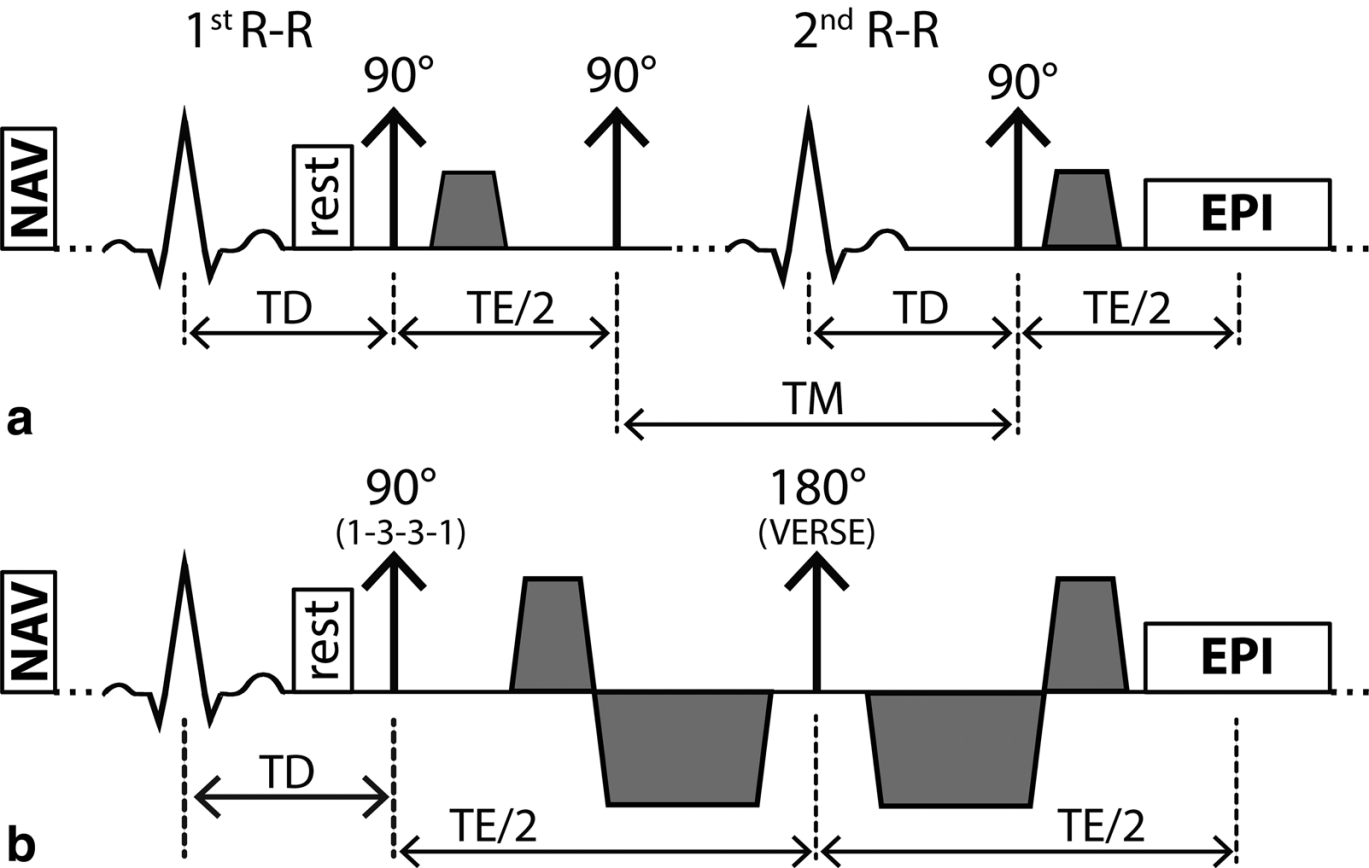
Sequence diagrams. STEAM acquisition spanning over two consecutive heartbeats (**a**) and SE acquisition with second order motion compensated diffusion encoding gradients including VERSE (variable rate selective excitation) echo pulse (**b**). Fat suppression is achieved by a binomial excitation pulse. The excitation slab is tilted with respect to the 90° or 180° pulses to allow for reduced field of view imaging. Before the first 90° excitation, rest slabs are applied orthogonal to the field of view in phase encoding direction to suppress residually excited signal. For both sequences, the trigger delay was set to mid systole and images were encoded by identical single‐shot EPI readouts.

Figure 1 illustrates the ECG‐triggered and diffusion weighted STEAM and SE sequences used in the present study. For the SE variant, second‐order motion compensated diffusion gradients are used 38.

The SNR of SE and STEAM depend on sequence timing parameters (echo time: TE, repetition time TR, mixing time TM), tissue properties (relaxation times T1, T2, diffusivity D), imaging parameters (voxel size ΔV, number of signal averages NSA, flip angle α), and diffusion encoding strength b. Here SNR efficiency (i.e., SNR per unit time) for the SE and STEAM sequences is defined as:

(1) $$SNR_{t}(SE)\propto \frac{1-e^{-TR/T1}}{1-\text{cos}\alpha e^{-TR/T1}}\text{sin}\alpha e^{-TE_{SE}/T2}e^{-b\cdot D}\frac{\Delta V}{\sqrt{NSA}}$$

(2) $$SNR_{t}(STEAM)\propto \frac{1}{2}\frac{1-e^{-TR/T1}}{1-\text{cos}\alpha e^{-TR/T1}}\text{sin}\alpha e^{-TM/T1}e^{-TE_{STEAM}/T2}e^{-b\cdot D}\frac{\Delta V}{\sqrt{NSA}\sqrt{2}}$$

In Eq. (2), the factors 1/2 and $1/\sqrt{2}$ account for the inherent signal loss in STEAM and the fact that two cardiac cycles are required to encode the stimulated echo. Because of ECG triggering, TR and TM are determined by the subject's heart rate (HR) according to:

(3) TR=60​  min−1HR[min−1]×1000 [ms] TM=TR−TESTEAM2.

**Figure 2:**
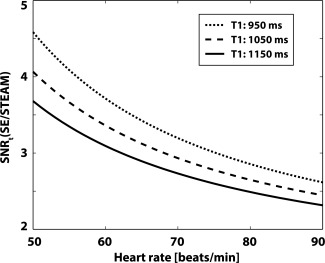
Theoretical SNR efficiency. The SNR efficiency ratio of SE versus STEAM is seen to decrease with increasing heart rate. At a heart rate of 60 min^‐1^, b‐value = 450 s/mm^2^, TR/TE = 1000 ms/70 ms (SE), TR/TM/TE = 1000 ms/985/31 ms (STEAM) the relative gain in SNR efficiency of SE versus STEAM is 3.5×.

In Figure 2, the ratio of SNR efficiency for SE versus STEAM, SNR_t_(SE/STEAM), is presented with sequence parameters according to the gradient system used in this study (b‐value = 450 s/mm^2^, TE_SE_ = 70 ms, TE_STEAM_ = 31 ms). The SNR_t_(SE/STEAM) ratio is seen to decrease with increasing heart rate but remains greater than 2.3 up to heart rates of 90 min^−1^. At a heart rate of 60 min^−1^ and T_1_/T_2_ = 1030/52 ms 49, 50, the theoretical SNR gain of SE relative to STEAM is approximately 3.5×.

### Study Protocol

Cardiac SE and STEAM diffusion weighted imaging were implemented on a 1.5 Tesla (T) Philips Achieva System (Philips Healthcare, Best, The Netherlands) with gradients delivering 80 mT/m maximum strength at a slew rate of 100 mT/m/ms per physical axis. Signal was received with a five‐channel cardiac receiver array. Written informed consent was obtained from all subjects before imaging. The study protocol was approved by the ethics committee of the Canton of Zurich. Consent included imaging as well as publication of anonymized data.

### Phantom Measurements

A phantom with isotropic diffusivity was made from an aqueous agar solution with a concentration of 40 g/L. T1 was reduced by addition of 2.5 × 10^−6^ mmol/L Gadolinium (Primovist, Bayer Schering, Germany) and relaxation times determined by a vendor preimplemented imaging sequence 51: T1_Agar_ = 1153 ± 10 ms, T2_Agar_ = 49.6 ± 0.4 ms within a region of interest inside the phantom.

To probe differences of the diffusion process during SE and STEAM diffusion encoding, a dedicated diffusion phantom with fiber structures mimicking cardiac myofibers was used in a second set of phantom measurements. The phantom consists of two crossing fiber bundles (∼20 mm diameter) of hydrophobic polyethylene cords as described previously 52.

The SNR efficiency of SE and STEAM was determined for several b‐values (50, 100, 200, 300, and 450 s/mm^2^). Diffusion weighted images were acquired with in‐plane resolution: 2.8 × 2.8 mm^2^, slice thickness: 16 mm (isotropic phantom)/12 mm (anisotropic phantom), field of view (FOV): 230 × 98 mm^2^, TR/TE (SE): 1000 ms/70 ms, TR/TE (STEAM): 1000 ms/31 ms, flip angle (SE, STEAM): 90°, four signal averages and six diffusion encoding directions (Table 1). To measure noise, the scans were repeated without RF and gradient pulses keeping the receiver gain and bandwidth the same as for actual imaging. Sufficient time (>10 s) was allowed between image and noise acquisition to ensure complete signal decay. SNR was determined for each voxel by dividing the absolute signal value of the diffusion weighted images by the standard deviation of the real part of complex noise in a local mask of 9 by 9 pixels. SNR_t_(SE/STEAM) of the anisotropic diffusion phantom was corrected for different T1/T2 values between both phantoms (T1_diffusion phantom_ = 934 ± 42 ms, T2_diffusion phantom_ = 104.2 ± 4 ms).

**Table 1:** Imaging Parameters of SNR and DTI Phantom Experiments

	Resolution	b‐values [s/mm^2^]	Flip angle [deg]	No. of directions	NSA (per b‐value/dir.)
SNR measurement (isotropic phantom)					
SE	2.8 × 2.8 × 16mm^3^	50,100,200,300,450	90	6	4
STEAM	2.8 × 2.8 × 16mm^3^	50,100,200,300,450	90	6	4
SNR measurement (anisotropic phantom)				
SE	2.8 × 2.8 × 12mm^3^	50,100,200,300,450	90	6	4
STEAM	2.8 × 2.8 × 12mm^3^	50,100,200,300,450	90	6	4
DTI measurement (anisotropic phantom)					
SE	2.8 × 2.8 × 12mm^3^	100,450	16	12	16
STEAM	2.8 × 2.8 × 12mm^3^	100,450	90	12	16

DTI was performed without the b = 0 s/mm^2^ image. Instead, three low diffusion weighted images (b = 100 s/mm^2^) were acquired along orthogonal directions along with nine additional diffusion weighted images (b = 450 s/mm^2^) distributed on the edge of a cube to maximize gradient usage and hence gradient strength. The duration of diffusion encoding gradients for the different diffusion weightings was kept the same and differences in b‐values were achieved by scaling the gradient strength. Imaging parameters were as follows: in‐plane resolution: 2.8 × 2.8 mm^2^, slice thickness: 12 mm, FOV: 230 × 98 mm^2^, TR/TE (SE): 1000 ms/70 ms, TR/TE (STEAM): 1000 ms/31 ms, number of signal averages: 16. To examine the effect of the sequence upon MD and FA, rather than any effects due to intrinsic SNR dependence 53, the flip angle of the SE excitation pulse was reduced to 16° to match SNR of the corresponding STEAM sequence (flip angle STEAM: 90°). Hence, variations in MD and FA can be uniquely assigned to differences during the diffusion encoding process.

### In Vivo Measurements

Data were acquired in seven healthy subjects without history of cardiac disease (five female, weight 64 ± 6 kg, age 28 ± 6 years, heart rate 64 ± 10 beats/min, min/max heart rates: 49/89 beats/min). Before diffusion imaging, cine data with a temporal resolution of 10 ms were acquired in two chamber and short axis view orientations. According to the cine images, systolic quiescent time points were determined on a per subject basis with a mean delay of 316 ± 19 ms.

Diffusion weighted imaging was performed during breath‐holding in short‐axis view orientation with a reduced field‐of‐view (FOV) technique 54. Consistent levels of breath‐holds were ensured by the use of a respiratory navigator placed on the right hemi diaphragm with a gating window of 5 mm. To avoid aliasing from residual excitation along the phase encoding direction, saturation slabs orthogonal to the imaging plane were played out before the RF excitation pulse (see Figure 1). A 1‐3‐3‐1 binomial spatial‐spectral excitation pulse for fat suppression 55 was used in the SE case. The duration of the 180° refocusing pulse was minimized using variable rate selective excitation (VERSE) 56 (Fig. 1). Diffusion weighting was performed by unipolar gradients (STEAM) and second order motion compensated gradient waveforms (SE) as proposed in 38, 42.

To minimize the effects of myocardial strain, the STEAM sequence was timed to the systolic strain “sweet spot” 15. The centers of mass of the STEAM and SE diffusion gradients within an R–R interval were aligned, resulting in a trigger delay for the SE sequence of 45% peak systolic contraction. In vivo SNR measurements were performed in each volunteer similar to the phantom experiments with a slice thickness of 16 mm to increase SNR. To guarantee identical b‐values for the SE and STEAM sequences, the effective b‐values during STEAM acquisitions were calculated based on the actual heart rates recorded during the in vivo experiments. Additionally, sufficient time to recover between the breath‐holds was ensured to avoid significant heart rate variations during scanning. To avoid magnetization transients during imaging, the first average was used as dummy scan and discarded.

For DTI, total scan time of the in vivo experiments was matched between SE and STEAM. Accordingly, 8 signal averages per diffusion encoding direction were acquired with STEAM, while 16 averages were recorded with SE (Table 2). The imaging slice (slice thickness: 8 mm) was positioned at a mid‐ventricular level. Data acquisition was split into multiple breath‐holds by acquiring all signal averages of a single diffusion encoding direction during a single breath‐hold. Between the breath‐holds, sufficient time for complete relaxation of magnetization was insured.

**Table 2:** Imaging Parameters of SNR and DTI In Vivo Experiments

	Resolution	b‐values [s/mm^2^]	Flip angle [deg]	No. of directions	NSA (per b‐value/dir.)
SNR measurement (in‐vivo)					
SE	2.8 × 2.8 × 16mm^3^	50,100,200,300,450	90	6	4
STEAM	2.8 × 2.8 × 16mm^3^	50,100,200,300,450	90	6	4
DTI measurement (in‐vivo)					
SE	2.8 × 2.8 × 8mm^3^	100,450	90	12	16
STEAM	2.8 × 2.8 × 8mm^3^	100,450	90	12	8

Additionally, DTI and SNR measurements were repeated during respiratory navigator‐gated free‐breathing acquisition with identical imaging and sequence parameters. Data collection was performed in a subgroup (n = 4, all female, weight 61 ± 10 kg, age 26 ± 2 years, heart rate 60 ± 8 beats/min, min/max heart rates: 44/71 beats/min) of the seven healthy volunteers in a separate imaging session. The methods and results are listed in the Appendix and Supporting Figure S1, which is available online.

### Data Analysis

The mean SNR of the phantom and in vivo data was determined for all acquired b‐values ranging from b = 50 s/mm^2^ to 450 s/mm^2^. SNR efficiency ratios of the SE sequence relative to the STEAM approach were calculated and compared with the theoretical values according to equations (1) and (2) taking the individual sequence timing (TR, TM, TE) into account. Relaxation times (T1 = 1030 ms/T2 = 52 ms) were taken from literature 49, 50.

For diffusion tensor analysis, images were first registered to the mean image using affine image transformations [elastix toolbox 57]. The in vivo SNR was determined using myocardial contours. To avoid partial voluming effects, voxels at the epi and endocardial borders were excluded from the statistics. The actual b‐values due to heart rate variations were corrected for by adjusting b_100_ and b_450_ to the corresponding true values and the b‐matrix modified for the proposed sampling scheme. The corresponding set of equations reads:

(4) $$B^{†}\vec{S}=\vec{D}$$

with the modified b‐matrix:

(5) $$B=\left[\begin{array}{l} b_{100}\left(\begin{array}{cccccc} x_{diff01}^{2} & y_{diff01}^{2} & z_{diff01}^{2} & 2xy_{diff01} & 2xz_{diff01} & 2yz_{diff01}\\ & & & \vdots & & \\ x_{diff03}^{2} & y_{diff03}^{2} & z_{diff03}^{2} & 2xy_{diff03} & 2xz_{diff03} & 2yz_{diff03} \end{array}\right)\left(\begin{array}{c} -1\\ -1\\ -1 \end{array}\right)\\ b_{450}\left(\begin{array}{cccccc} x_{diff04}^{2} & y_{diff04}^{2} & z_{diff04}^{2} & 2xy_{diff04} & 2xz_{diff04} & 2yz_{diff04}\\ & & & \vdots & & \\ x_{diff12}^{2} & y_{diff12}^{2} & z_{diff12}^{2} & 2xy_{diff12} & 2xz_{diff12} & 2yz_{diff12} \end{array}\right)\left(\begin{array}{c} -1\\ \vdots \\ -1 \end{array}\right) \end{array}\right]$$

b_100_ and b_450_ are the two heart rate adjusted b‐values (nominal values: 100 and 450 s/mm^2^). $\vec{S}$ denotes the negative logarithmic signal vector

(6) $$\vec{S}=-\text{ln}({\left[S_{\text{diff01}}\cdots S_{\text{diff12}}\right]}^{T})$$

The $\vec{D}$ vector contains the diffusion tensor elements and the b = 0 s/mm^2^ signal S_0_:

(7) $$\vec{D}={\left[D_{x^{2}}\;D_{y^{2}}\;D_{z^{2}}\;D_{\text{xy}}\;D_{\text{xz}}\;D_{\text{yz}}\;{\text{ln(}S}_{0})\right]}^{T}$$

†, *T* denote the Moore‐Penrose pseudo inverse and transpose, respectively.

Upon tensor calculation the helix and transverse angles were calculated. Here the helix angle captures the local helix elevation, i.e., the angle between the projection of the first eigenvector of the diffusion tensor onto the epicardial surface and the transmural plane. The transverse angle represents the deviation of the helix from circumferential structure, i.e., the angle between the first eigenvector projected onto the radial circumferential plane and the circumferential contour 58. For each diffusion tensor, a normalized transmural position was calculated. Angle analysis was performed for the anterior, septal, inferior and lateral region separately. Furthermore, the gradient of a linear fit to the transmural helix angle course was calculated. Reproducibility of MD and FA were assessed by a two‐tailed paired t‐test and the Bland‐Altman method 59.

## RESULTS

### SNR Measurements

**Figure 3:**
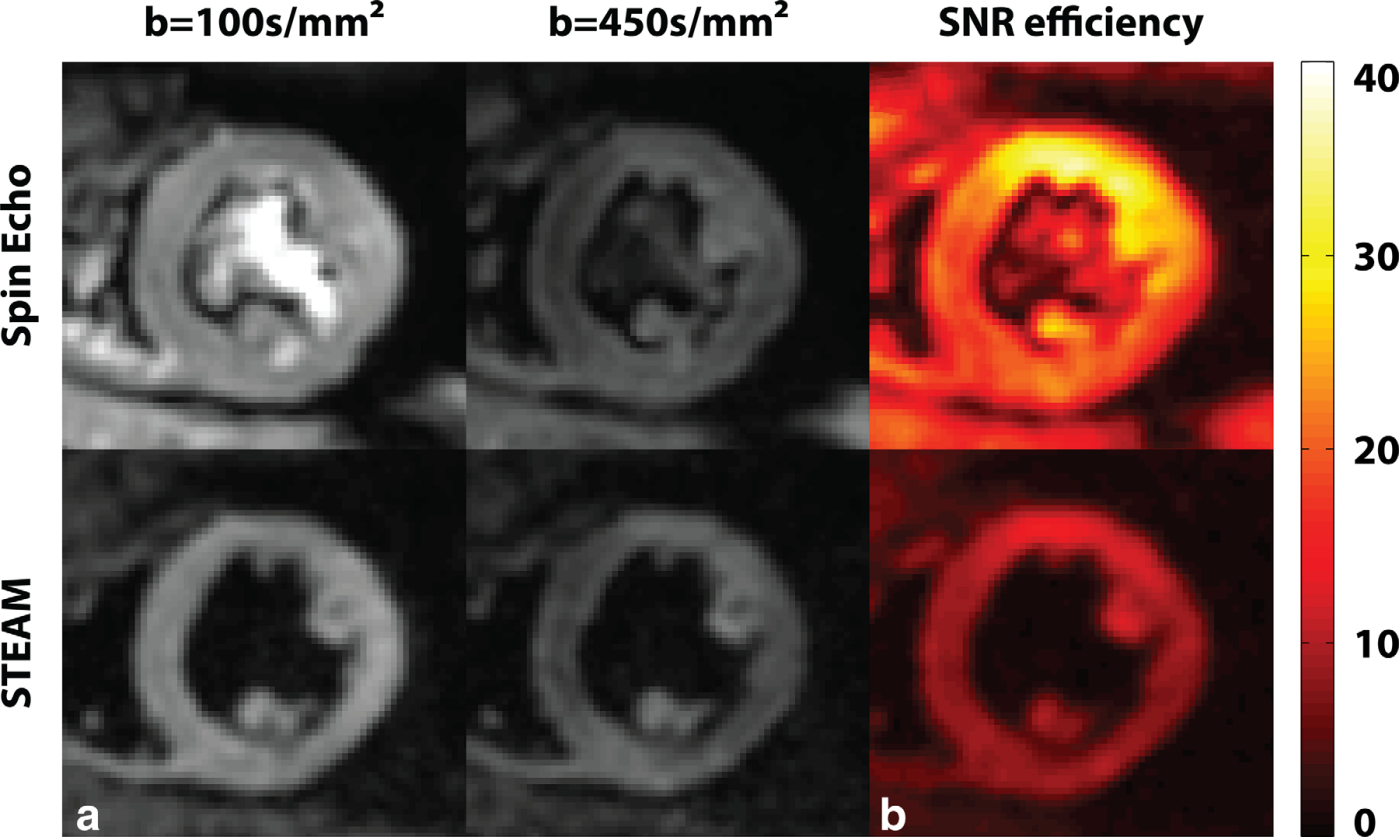
In vivo data. **a:** Example dataset acquired at b = 100 s/mm^2^ and 450 s/mm^2^. **b:** Corresponding SNR efficiency maps for b = 450 s/mm^2^.

Figure 3a shows example in vivo images for the b = 100 and 450 s/mm^2^ acquisitions obtained by the SE and STEAM approach. The bright blood pool signal in the b = 100 s/mm^2^ image of the SE measurements is dephased with increasing diffusion weighting. No signal contributions from blood in the STEAM case are noticeable. SNR efficiency maps of SE and STEAM for a single average obtained with a b‐value of 450 s/mm^2^ are compared in Figure 3b.

**Figure 4:**
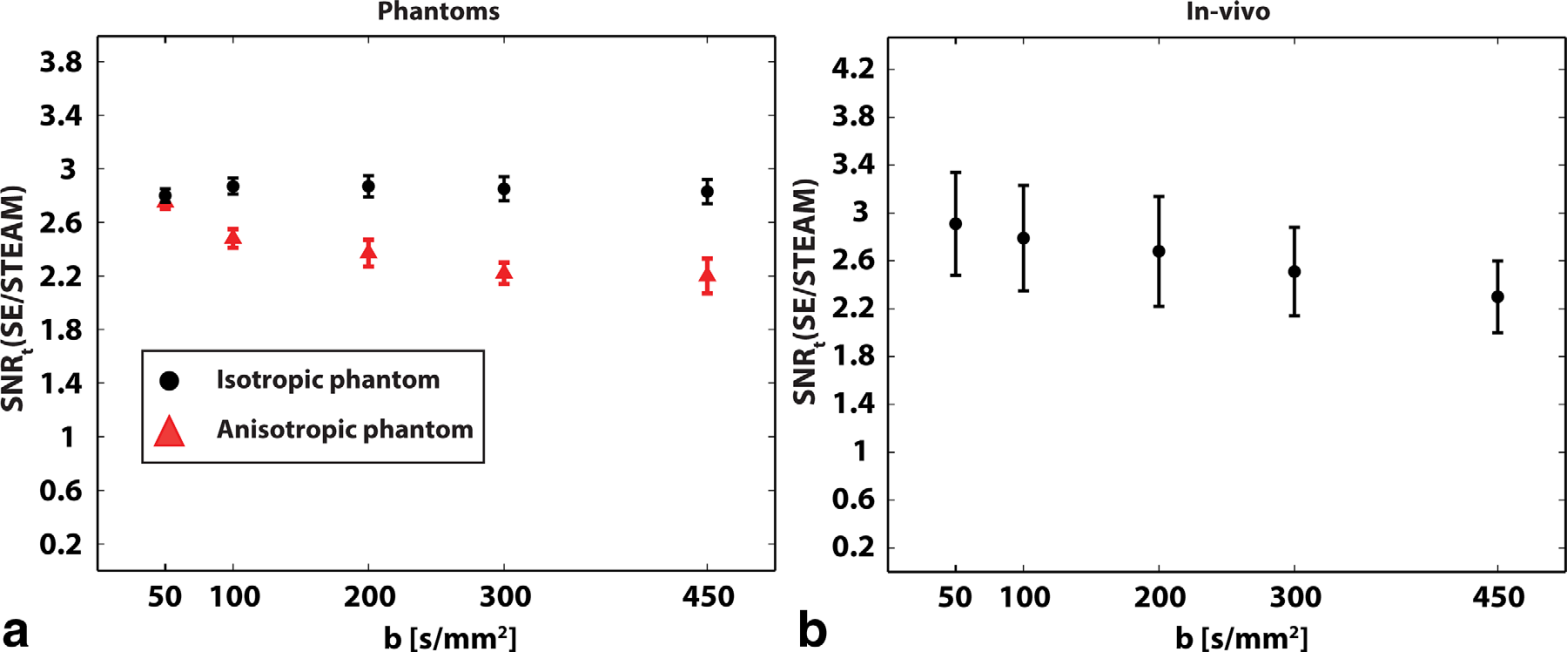
SNR efficiency ratios of isotropic agar and anisotropic diffusion phantom as well as of in vivo measurements are shown for b‐values ranging from 50 to 450 s/mm^2^. **a:** While the isotropic agar phantom shows no b‐value dependency, SNR_t_(SE/STEAM) is seen to decrease with increasing diffusion weighting in the anisotropic diffusion phantom. **b:** In vivo data reveal b‐value dependency of SNR_t_(SE/STEAM) similar to anisotropic diffusion phantom.

SNR efficiency ratios SNR_t_(SE/STEAM) per single average of the phantom and in vivo data are plotted in Figure 4. The data of the isotropic agar phantom yielded a mean ratio of 2.84 ± 0.08, which agrees well with theory (theoretical SNR_t_(SE/STEAM) = 3.07) as shown in Figure 4a. While SNR_t_(SE/STEAM) was independent of the b‐value in the isotropic phantom, a distinct decrease of SNR_t_(SE/STEAM) was measured in the anisotropic diffusion phantom. Here the ratio of SNR efficiency for SE versus STEAM was found to decrease from 2.75 ± 0.05 to 2.20 ± 0.13 when increasing the b‐value from 50 to 450 s/mm^2^. The mean SNR efficiencies for the phantom measurements are listed in Supporting Tables S1 and S2. Similarly, the in vivo data revealed a reduced SNR_t_(SE/STEAM) with increasing b‐value as shown in Figure 4b. The in vivo SNR efficiency ratios decreased from 2.91 ± 0.43 to 2.30 ± 0.30 when increasing the b‐value from 50 s/mm^2^ to 450 s/mm^2^ (theoretical SNR_t_(SE/STEAM) = 3.46 ± 0.45). The mean SNR efficiencies across all volunteers and b‐values are listed in Supporting Table S3.

### DTI Measurements

MD values obtained from SE data in the anisotropic diffusion phantom were found to be higher (1.58 ± 0.10 10^−3^mm^2^/s) when compared with STEAM (MD_STEAM_ = 1.14 ± 0.13 10^−3^mm^2^/s). Likewise FA values obtained with SE were lower (FA_SE_ = 0.29 ± 0.07) relative to STEAM (FA_STEAM_ = 0.53 ± 0.11).

In vivo DTI data quality was assessed by calculating the percentage of negative eigenvalues. While 0.02 ± 0.05% of the diffusion tensors derived from SE were found to have negative eigenvalues, STEAM resulted in 2.53 ± 1.63%.

**Figure 5:**
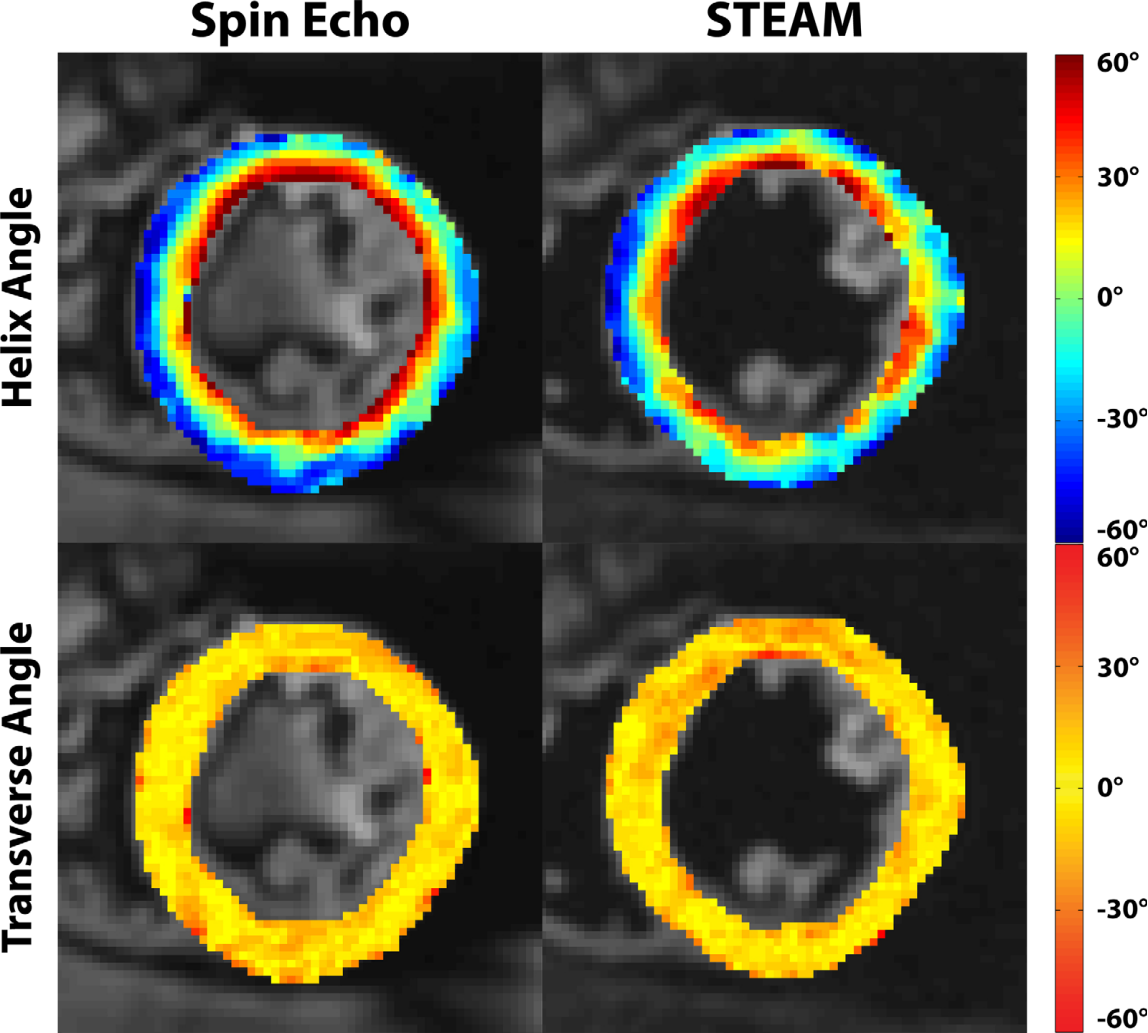
Helix and transverse angle maps. The linear decrease of helix angles from endo‐ to epicardium is visible, however more coherent in the SE case. Transverse angles are close to zero degrees for both sequences except for nonnegative values at the intersection of left and right ventricular structures and near the papillary muscles.

In Figure 5, example helix and transverse angle maps are compared for SE and STEAM. A clear progression in helix angle from positive to negative values from endo‐ to epicardium can be observed. Reduced SNR in the STEAM case, however, causes patches with increased angle variations.

**Figure 6:**
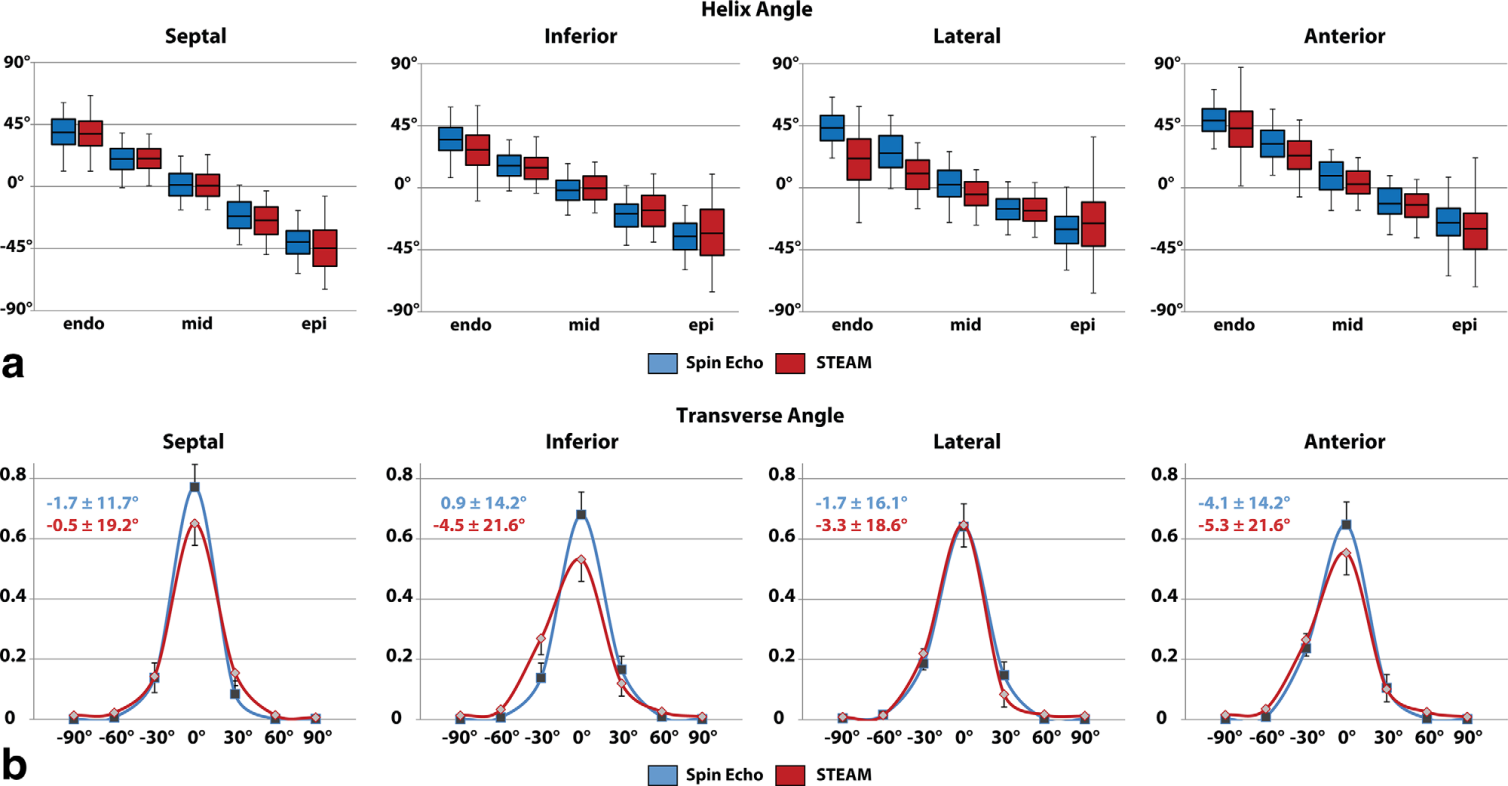
Sectorwise helix and transverse angle statistics. **a:** Comparison of transmural helix angle distribution for SE (blue) and STEAM (red) (solid box: 50% percentile, error bars: 90% percentile of the helix angle distribution in circumferential direction). Helix angle variations are more pronounced in STEAM, particularly at the endo‐ and epicardial region. **b:** Histograms of transverse angles show reduced dispersion of transverse angle for SE.

Statistics on helix and transverse angles across all volunteers for the anterior, septal, inferior and lateral segments are reported in Figure 6. The solid boxes and error bars correspond to the 50% and 90% percentiles of the helix angle distribution along the circumferential dimension. The linear dependency of the helix angles as a function of transmural depth is clearly evident for SE and STEAM. In the STEAM case, however, endo‐ and epicardial helix angles are found to be less steep with increased angle variation at the inferior–lateral region relative to SE. The root mean squared error (RMSE) of linear regression of the transmural helix angle distribution was found to be significantly reduced in SE versus STEAM (13.7 ± 2.6° versus 18.0 ± 2.8°; *P* < 0.01). The standard deviation of the transverse angles across all subjects and sectors was significantly smaller for SE compared with STEAM: (13.7 ± 1.2° versus 19.7 ± 2.0°; *P* < 0.01).

**Figure 7:**
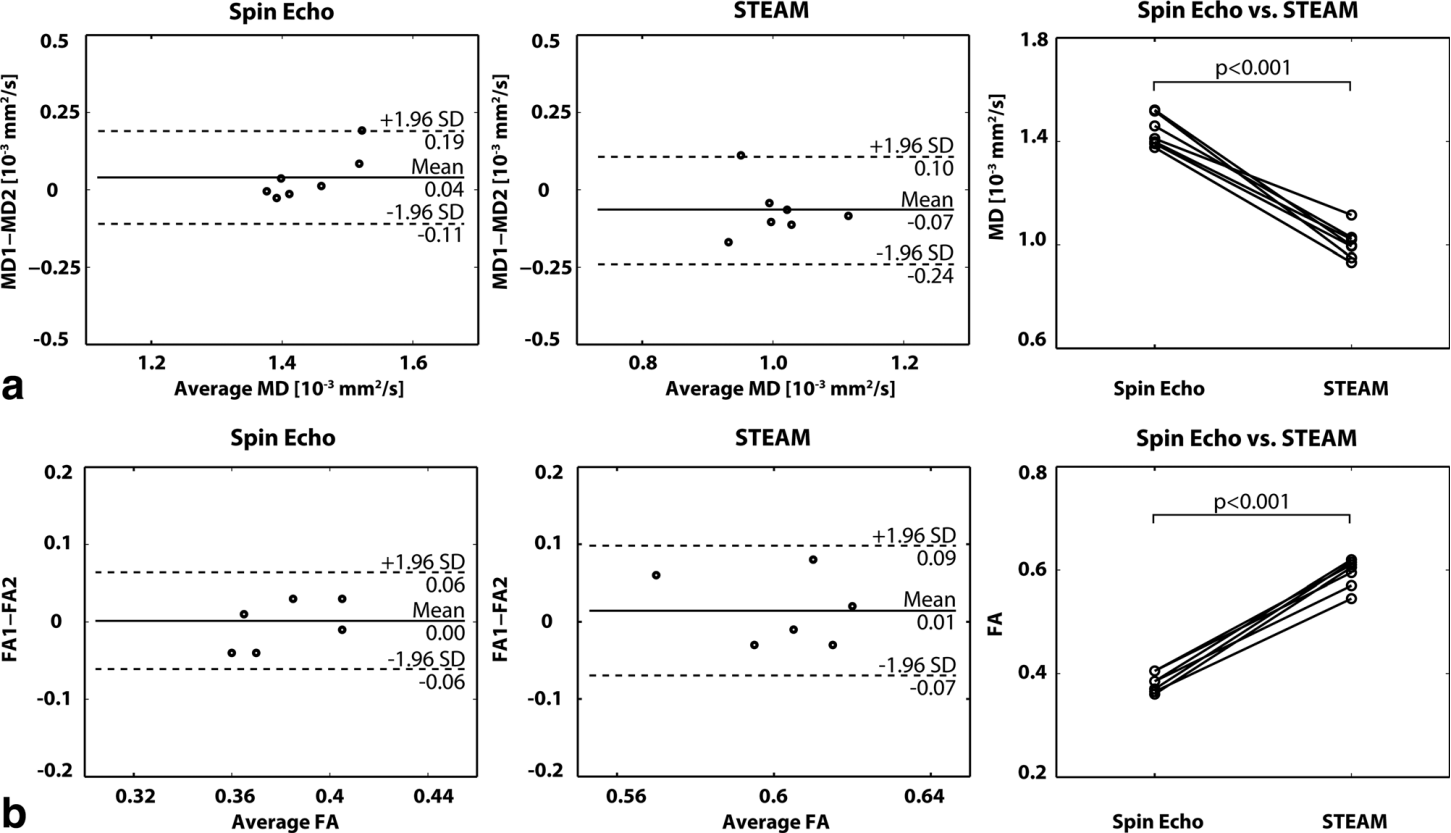
Bland‐Altman and line plots of MD (**a**) and FA (**b**) representing intra subject reproducibility of SE versus STEAM. No statistical differences were observed between repeat measurements for MD and FA for both SE and STEAM. Significant differences were found for MD and FA between both sequences.

The reproducibility of MD and FA was determined by repeated acquisitions during one session. The average MD values were 1.43 ± 0.06 10^‐3^mm^2^/s for SE and 1.05 ± 0.08 10^‐3^mm^2^/s for STEAM. No statistically significant differences were found between repeated experiments for both SE and STEAM (SE: *P* = 0.31, STEAM: *P* = 0.10). However, differences for MD between SE and STEAM were statistically significant (*P* < 0.001). Mean FA values over all volunteers were 0.38 ± 0.02 for SE and 0.59 ± 0.03 for STEAM with no statistically significant differences between repeated experiments (SE: *P* = 0.91, STEAM: *P* = 0.41) in each case. However, FA differences between the SE and STEAM were statistically significant (*P* < 0.001). Corresponding Bland‐Altman and line plots for the in vivo MD and FA values are shown in Figure 7.

**Figure 8:**
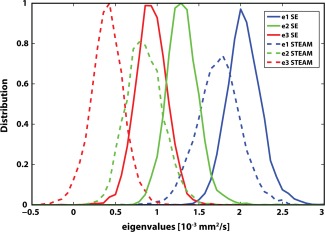
Eigenvalue analysis. Diffusion tensor eigenvalue (e1, e2, e3) histograms for SE (solid line) and STEAM (dashed line). SE eigenvalues show a distinct and dense distribution, while STEAM eigenvalue histograms are broadened.

Figure 8 shows the distribution of the first, second and third eigenvectors (e1, e2, e3) for SE and STEAM DTI data across all volunteers. The SE distributions show narrow, almost equally high distinct peaks, whereas the eigenvalue spectrum in the STEAM case is broadened. The small percentage of negative eigenvalues (e3) in the STEAM case can be appreciated as well. Furthermore, the maximums of the SE eigenvalue distributions are increased and less separate relative to STEAM, in agreement with the results for MD and FA.

## DISCUSSION

In this study the relative SNR gain of cardiac‐triggered, motion‐compensated SE diffusion tensor imaging with respect to STEAM has been demonstrated using both phantom and in vivo data. The SNR gain was found to improve the accuracy of diffusion metrics including helix and transverse angle maps.

At a b‐value of 50 s/mm^2^ the measured SNR efficiency gain of 2.84 of SE versus STEAM agreed well with theory in the phantoms. In vivo, the measured gain in SNR efficiency at the lowest b‐value was smaller compared with theory [2.91 ± 0.21 (in vivo) versus 3.46 ± 0.45 (theory)]. This is attributed in parts to slice profile imperfections of the VERSE echo pulse 56 and partial volume effects through‐slice in vivo. All SNR measurements were obtained from a single signal average. Thereby confounding factors due to image misregistration and phase correction for averaging of complex data were avoided. To facilitate these measurements, the slice thickness was increased to 16 mm contributing to increased partial voluming.

While the ratio of SNR efficiency of SE versus STEAM was found to be independent of the b‐value in the isotropic agar phantom, decreasing values were measured with increasing b‐value for both the anisotropic diffusion phantom and in the in vivo myocardium. This result is explained by differences in diffusion times ΔT (ΔT_STEAM_ = 1000 ms versus ΔT_SE_ = 25 ms) 60, 61. Assuming a mean diffusivity of 1.4 × 10^−3^mm^2^/s and diffusion times of 25 ms for SE and 1000 ms for STEAM, the diffusion length is approximately 15 and 90 μm, respectively. In comparison, the thickness of cardiac myocytes is in the range of approximately 10–20 μm 62, 63. Hence, diffusion becomes restricted during STEAM encoding and diffusion induced phase distributions deviate from a Gaussian shape 64. The phase distribution in STEAM is expected to be stretched out along the fiber direction due to the long diffusion time and the lateral confinement by myofibers.

DTI data were acquired with a modified sampling method. Instead of acquiring a b = 0 s/mm^2^ image, three directions with a b‐value of 100 s/mm^2^ and nine directions with a b‐value of 450 s/mm^2^ were sampled. Thereby the effect of in vivo perfusion was reduced 65, 66, 67. Helix angle maps obtained with SE and STEAM revealed the expected linear decrease from endo‐ to epicardium in accordance with previous studies 13, 16, 18, 21, 23. However, STEAM data resulted in increased deviation from the linear function when compared with SE, in particular in the inferior–lateral region, which corresponds to areas of low SNR in the SNR efficiency maps. The drop in SNR is related to the distal positon of the inferior–lateral segment to the surface coils.

In this region of low SNR, the helix angle range was found to be underestimated compared with SE. Transverse angles were measured close to zero degrees on average, describing the circumferential alignment of myofibers. The lower SNR of STEAM, however, caused a larger variation around zero when compared with SE.

In vivo results for MD and FA were reproducible and in accordance with literature values of the in vivo human heart 13, 16, 18, 21, 23, 38. Significant differences in MD and FA were seen between in vivo STEAM and SE similar to previous results found in muscle tissue 60. The measurements in the anisotropic diffusion phantom confirmed these findings. While MD is higher in the SE case, FA is increased with STEAM. Increased fractional anisotropy FA_STEAM_ is represented by a broad separation of the eigenvalue distributions compared with a compact, distinct distribution pattern in the SE case.

By design, the total scan duration for breath‐held SE and STEAM imaging was kept identical in this study. However, SE acquisitions are favored for time‐efficient free‐breathing acquisitions as demonstrated in the supplemental material. In contrast, free‐breathing STEAM imaging requires a very narrow respiratory gating window between the second (decoding) and first (encoding) heartbeat 18 hence reducing scan efficiency significantly relative to SE. As demonstrated in the supplemental material, SE resulted in 30% increased scan efficiency compared with STEAM while SNR and DTI results were in very good agreement with the findings from the breath‐hold measurements.

Acceleration compensated spin‐echo cardiac DTI requires a high‐performance gradient system to reduce TE to approximately 65–70 ms for a b‐value of 450 s/mm^2^. With the recent introduction of clinical MR machines with high‐performance gradient systems, this requirement is expected to be met increasingly in the near future.

## CONCLUSIONS

Cardiac diffusion tensor imaging using motion‐compensated SE yields up to 2.9× increase in SNR efficiency relative to STEAM, which in turn translates to reduced deviation of helix and transverse angles from expected in vivo configurations. The SE method hence presents an attractive alternative to STEAM based approaches for cardiac diffusion tensor imaging of the in vivo heart on modern MR systems with high‐performance gradients.

## Supporting information


**Supporting Table S1.** Results of SNR efficiency of SE versus STEAM measured in isotropic agar phantom for different b‐values.
**Supporting Table S2.** Results of SNR efficiency measured in anisotropic diffusion phantom.
**Supporting Table S3.** Results of SNR efficiency of SE versus STEAM measured in vivo for different b‐values.
**Supporting Figure S1.** Free breathing acquisition: a) Helix and transverse angle maps. Similar to the breath hold case, the decrease of helix angles from endo‐ to epicardium is more coherent for SE. Transverse angles are close to zero degrees. b) Helix and transverse angle statistics: Comparison of transmural helix angle distribution for SE (blue) and STEAM (red) (solid box: 50% percentile, error bars: 90% percentile of the helix angle distribution in circumferential direction).

## Appendix

Because the SE approach requires only one cardiac cycle to acquire a diffusion weighted image (in contrast to two R–R intervals for STEAM), the sequence is particularly suited for free‐breathing navigator gated acquisition.

DTI acquisition was performed during free‐breathing with a navigator gating scheme as proposed by Nielles‐Vallespin et al 18 along three (b = 100 s/mm^2^) and nine (b = 450 s/mm^2^) diffusion directions without visual feedback system. Images were accepted if the first navigator of the STEAM sequence was within the acceptance window of 5 mm and the second navigator had a relative displacement of less than ±0.5 mm to the first one. The imaging parameters were identical to the parameters used for breath‐held imaging: resolution 2.8 × 2.8 mm^2^, slice thickness 8 mm, FOV: 230 × 98 mm^2^, TE/TR(SE): 70 ms/1‐R‐R, TE/TR(STEAM): 31 ms/2‐R‐R, signal averages SE/STEAM: 16/8. Imaging was timed to the systolic strain “sweet spot” 15. DTI analysis was performed based on mean diffusivity (MD), fractional anisotropy (FA), helix and transverse angles, the percentage of negative eigenvalues of the diffusion tensor and scan time efficiency. SNR was measured in a separate scan for b = 50 and 450 s/mm^2^ along six diffusion directions (16 mm slice thickness), four signal averages. DTI data reproducibility was not part of this sub‐study.

SE based DTI data acquisition was 30% faster than STEAM for all volunteers (7:06 ± 3:09 min:s versus 9:52 ± 2:25 min:s). Supplementary Figure S1a shows an example helix and transverse angle map. Similar to the breath‐hold acquisition (Fig. 5), the helix map for STEAM shows patches with increased angle variations and less steep angles endo‐ and epicardially. A linear transmural change of helix angles can be seen in Supplementary Figure S1b. Transverse angles were close to zero degrees with wider spread in the STEAM (‐1.2 ± 18.7)° case compared with SE (−2.2 ± 14.5)°. The RMSE of linear regression of the transmural helix angle distribution was reduced in all cases for SE versus STEAM (14.0 ± 0.7° versus 17.1 ± 1.5°). While 3.7 ± 1.7% of the diffusion tensors derived from STEAM had negative eigenvalues, no negative eigenvalue was found in the SE case. The average MD values were 1.48 ± 0.11 10^‐3^mm^2^/s for SE and 0.99 ± 0.06 10^−3^mm^2^/s for STEAM. Mean FA values over all volunteers were 0.37 ± 0.04 for SE and 0.61 ± 0.03 for STEAM. MD and FA were in accordance with breath‐hold and literature values 13, 16, 18, 21, 23, 38.

Similar to the breath‐hold measurements, the in vivo data show a SNR_t_(SE/STEAM) of 3.12 ± 0.66 to 2.79 ± 0.51 with increasing b‐value from 50 s/mm^2^ to 450 s/mm^2^ (theoretical SNR_t_(SE/STEAM) = 3.73 ± 0.67).

In summary, free‐breathing DTI and SNR results are in very good agreement with the results from breath‐hold acquisitions of this work and similar to previous studies 18, 23. In addition, scan time efficiency was significantly improved for SE compared with STEAM. Hence, besides the SNR benefit, free‐breathing navigator gating efficiency is increased when using SE based DTI.
