# Supplementary material for: Spin echo versus stimulated echo diffusion tensor imaging of the in vivo human heart
Source: Magn Reson Med. 2015 Oct 7;76(3):862–72. doi: 10.1002/mrm.25998 (PMC4989478; doi:10.1002/mrm.25998)
Supplement: Supplementary file 1 — Supporting Table S1. Results of SNR efficiency of SE versus STEAM measured in isotropic agar phantom for different b‐values. Supporting Table S2. Results of SNR efficiency measured in anisotropic diffusion phantom. Supporting Table S3. Results of SNR efficiency of SE versus STEAM measured in vivo for different b‐values. Supporting Figure S1. Free breathing acquisition: a) Helix and transverse angle maps. Similar to the breath hold case, the decrease of helix angles from endo‐ to epicardium is more coherent for SE. Transverse angles are close to zero degrees. b) Helix and transverse angle statistics: Comparison of transmural helix angle distribution for SE (blue) and STEAM (red) (solid box: 50% percentile, error bars: 90% percentile of the helix angle distribution in circumferential direction). [file MRM-76-862-s001.docx]

**Supporting information:**

| b [s/mm^2^] | SNR_t_(SE) | SNR_t_(STEAM) | SNR_t_(SE/STEAM) |
| --- | --- | --- | --- |
| 50 | 49.19±0.35 | 17.59±0.28 | 2.80±0.05 |
| 100 | 43.81±0.39 | 15.27±0.31 | 2.87±0.06 |
| 200 | 36.18±0.49 | 12.61±0.30 | 2.87±0.08 |
| 300 | 28.76±0.37 | 10.08±0.29 | 2.85±0.09 |
| 450 | 20.86±0.32 | 7.37±0.21 | 2.83±0.09 |

Supporting Table S1: Results of SNR efficiency of SE versus STEAM measured in isotropic agar phantom for different b-values.

| b [s/mm^2^] | SNR_t_(SE) | SNR_t_(STEAM) | SNR_t_(SE/STEAM) |
| --- | --- | --- | --- |
| 50 | 9.40±0.12 | 3.42±0.06 | 2.75±0.05 |
| 100 | 7.89±0.27 | 3.18±0.05 | 2.48±0.07 |
| 200 | 6.52±0.31 | 2.75±0.14 | 2.37±0.10 |
| 300 | 5.59±0.44 | 2.52±0.15 | 2.22±0.08 |
| 450 | 4.49±0.61 | 2.04±0.23 | 2.20±0.13 |

Supporting Table S2: Results of SNR efficiency measured in anisotropic diffusion phantom.

| b [s/mm^2^] | SNR_t_(SE) | SNR_t_(STEAM) | SNR_t_(SE/STEAM) |
| --- | --- | --- | --- |
| 50 | 44.29±5.78 | 15.40±2.20 | 2.91±0.43 |
| 100 | 39.10±5.89 | 14.16±1.92 | 2.79±0.44 |
| 200 | 33.51±5.19 | 12.64±1.94 | 2.68±0.46 |
| 300 | 27.63±3.24 | 11.17±1.61 | 2.51±0.37 |
| 450 | 22.16±2.81 | 9.67±1.07 | 2.30±0.30 |

Supporting Table S3: Results of SNR efficiency of SE versus STEAM measured in vivo for different b-values.


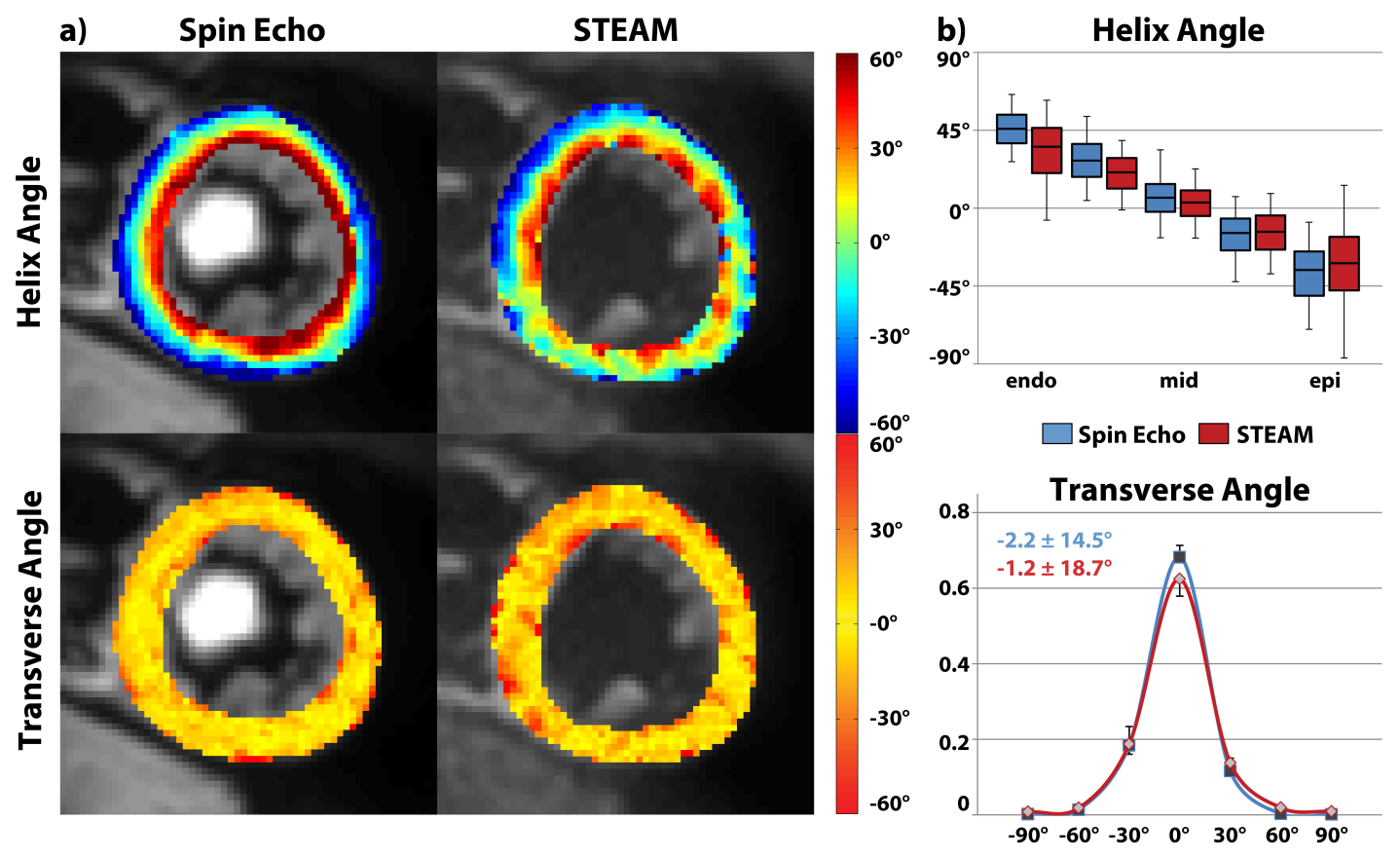
Supporting Figure S1: Free breathing acquisition: a) Helix and transverse angle maps. Similar to the breath hold case, the decrease of helix angles from endo- to epicardium is more coherent for SE. Transverse angles are close to zero degrees. b) Helix and transverse angle statistics: Comparison of transmural helix angle distribution for SE (blue) and STEAM (red) (solid box: 50% percentile, error bars: 90% percentile of the helix angle distribution in circumferential direction).
